# Perinatal Exposure to Insecticide Methamidophos Suppressed Production of Proinflammatory Cytokines Responding to Virus Infection in Lung Tissues in Mice

**DOI:** 10.1155/2013/151807

**Published:** 2013-12-03

**Authors:** Wataru Watanabe, Hiroki Yoshida, Akihiko Hirose, Toshi Akashi, Tomomi Takeshita, Nao Kuroki, Asami Shibata, Satoko Hongo, Seiko Hashiguchi, Katsuhiko Konno, Masahiko Kurokawa

**Affiliations:** ^1^Department of Microbiology, Graduate School of Clinical Pharmacy, Kyushu University of Health and Welfare, Yoshino 1714-1, Nobeoka, Miyazaki 882-8508, Japan; ^2^Department of Biochemistry, Graduate School of Clinical Pharmacy, Kyushu University of Health and Welfare, Yoshino 1714-1, Nobeoka, Miyazaki 882-8508, Japan; ^3^Division of Risk Assessment, Biological Safety Research Center, National Institute of Health Sciences, 1-18-1, Kamiyoga, Setagaya-ku, Tokyo 158-8501, Japan; ^4^Department of Microbiology and Infectious Diseases, School of Pharmaceutical Sciences, Kyushu University of Health and Welfare, Yoshino 1714-1, Nobeoka, Miyazaki 882-8508, Japan

## Abstract

Methamidophos, a representative organophosphate insecticide, is regulated because of its severe neurotoxicity, but it is suspected of contaminating agricultural foods in many countries due to illicit use. To reveal unknown effects of methamidophos on human health, we evaluated the developmental immunotoxicity of methamidophos using a respiratory syncytial virus (RSV) infection mouse model. Pregnant mice were exposed to methamidophos (10 or 20 ppm) in their drinking water from gestation day 10 to weaning on postnatal day 21. Offsprings born to these dams were intranasally infected with RSV. The levels of interleukin-6 (IL-6) and interferon-gamma in the bronchoalveolar lavage fluids after infection were significantly decreased in offspring mice exposed to methamidophos. Treatment with methamidophos did not affect the pulmonary viral titers but suppressed moderately the inflammation of lung tissues of RSV-infected offspring, histopathologically. DNA microarray analysis revealed that gene expression of the cytokines in the lungs of offspring mice exposed to 20 ppm of methamidophos was apparently suppressed compared with the control. Methamidophos did not suppress IL-6 production in RSV-infected J774.1 cell cultures. Thus, exposure of the mother to methamidophos during pregnancy and nursing was suggested to cause an irregular immune response in the lung tissues in the offspring mice.

## 1. Introduction

Methamidophos, an organophosphate insecticide, is a well-known toxicant, and its usage is restricted during planting of vegetables [1]. Recently, it was reported that the levels of methamidophos in horticultural greenhouse workers continuously exposed to it were similar to those who ingested methamidophos-contaminated food [2]. A representative mechanism of action of methamidophos is inhibition of cholinesterase in the central nervous system, but delayed neuropathy is speculated to be due to no association with acetylcholinesterase [3]. Toxic effects of methamidophos on male reproductive organs were also reported [4]. However, except for those on the central nervous system, the cumulative effects of exposure to methamidophos, either directly or in utero, on living organism have not been elucidated clearly. To protect mothers and children from environmental contaminants including organophosphate insecticides, the developmental immunotoxicity of methamidophos should be strictly assessed.

A novel assay system for evaluating the developmental immunotoxicity of environmental contaminants, brominated flame retardants (BFRs), using a mouse model infected with respiratory syncytial virus (RSV) has been established and reported previously [5]. RSV, a member of the family *Paramyxoviridae*, is the most prevalent agent of acute lower respiratory infections in infants and young children [6]. Because it was reported that clinically severe RSV infection is seen primarily in young children with naïve immune systems and/or genetic predispositions [7], patients with suppressed T-cell immunity [6], and the elderly [8], we applied the virus to an assay system evaluating the immunotoxicity of BFRs. Perinatal exposure to a representative BFR, decabrominated diphenyl ether (DBDE), was previously shown to increase viral titers in the lungs of RSV-infected offspring mice on day 5 after infection, resulting in exacerbation of pneumonia [9]. Moreover, perinatal exposure to DBDE was suggested to cause a functional disorder of the primary immunity responding to RSV infection [10].

In this study, we measured several parameters associated with immune response and/or inflammation in lung tissues of RSV-infected offspring to evaluate effects of maternal and postnatal exposure to methamidophos on the immune system in a murine model. Perinatal exposure to methamidophos was shown to suppress production of the specific cytokines responding to RSV infection in the lung tissues in the offspring mice.

## 2. Materials and Methods

### 2.1. Animals

Female (6 weeks old) and male (8 weeks old) BALB/c mice was purchased from Kyudo Animal Laboratory (Kumamoto, Japan) and housed at 25 ± 2°C. The mice were allowed free access to a conventional solid diet, CRF-1 (Oriental Yeast Co., Chiba, Japan), and water and used in this experiment after 7 d acclimation. The animal experimentation guideline of the Kyushu University of Health and Welfare were followed in the animal studies.

### 2.2. Cells and Virus

The mouse macrophage cell line J774.1 (JCRB0018) was purchased from JCRB Cell Bank (Ibaraki, Japan) and maintained in Roswell Park Memorial Institute (RPMI)-1640 medium supplemented with heat-inactivated 10% fetal calf serum. The A2 strain of RSV was obtained from American Type Culture Collection (ATCC, Rockville, MD, USA) and grown in HEp-2 cell (human epidermoid carcinoma, ATCC CCL-23) cultures.

### 2.3. Chemical Compound

Methamidophos was purchased from Sigma-Aldrich Japan (Tokyo, Japan). Methamidophos was completely dissolved in tap water.

### 2.4. Perinatal Exposure to Methamidophos

Seven-week-old female mice and 9-week-old male mice were paired for 3 d. The day of conception was determined by observation of a vaginal plug in the female. At 3 d after conception, the females were randomly divided into three groups for methamidophos exposure at 0, 10, or 20 ppm. Each group was composed of six mice. These mice continuously ingested methamidophos in drinking water from 10 days after conception to weaning on postnatal day 21. After weaning, offspring mice were given the CRF-1 diet and tap water. On postnatal day 28, offspring mice in each group were used for an RSV infection test. Throughout these experiments, both chows and drinking water were given ad libitum, and the consumption was checked weekly.

### 2.5. RSV Infection Test

An RSV infection test was performed according to our previous report [5]. Briefly, four-week-old offspring mice were infected intranasally with 5 × 10^6^ PFU of the A2 strain of RSV under anesthesia. In a mock-infected group, offspring mice were given phosphate-buffered saline (PBS) intranasally. On day 1 or 5 after infection, bronchoalveolar lavage fluid (BALF) was obtained from the mice under anesthesia by instilling 1.0 mL of cold PBS into the lungs and aspirating it from the trachea using a tracheal cannula. For a DNA microarray test of the lungs, mock- or RSV-infected mice were sacrificed by cervical dislocation on day 1 after infection, and the lungs were removed and placed in RNAlater reagent (Qiagen, Germany) and stored at 4°C until use.

For histological examination of the infected lungs, RSV-infected mice were sacrificed by cervical dislocation on day 5 after infection, and the lungs were removed and placed in buffered formalin for a minimum of 24 h. The tissue was then embedded in low-melting point paraffin, sectioned at a thickness of 5 *μ*m, and stained with hematoxylin and eosin.

### 2.6. Enzyme-Linked Immunosorbent Assays

Interleukin (IL)-1*β*, IL-4, IL-6, IL-10, interferon (IFN)-*γ*, and tumor necrosis factor (TNF)-*α* levels in BALF were measured using specific ELISA kits (Ready-set-go, eBioscience Inc., San Diego, CA, USA) according to the manufacturer's instructions. IL-12 levels in BALF were also measured using a specific kit (Ready-set-go, eBioscience Inc.) for IL-12 p70, without interference by the p40 monomer or the related protein IL-23, according to the manufacturer's instructions. Levels of colony stimulating factor 3 (G-CSF) in BALF were measured using specific ELISA kit (Quantikine, R&D Systems, Inc., Minneapolis, MN, USA) according to the manufacturer's instructions. The lower limits of detection of the kits are 8 (pg/mL) for IL-1*β*, 4 (pg/mL) for IL-4, 4 (pg/mL) for IL-6, 8 (pg/mL) for IL-10, 15 (pg/mL) for IL-12 p70, 4 (pg/mL) for IFN-*γ*, 8 (pg/mL) for TNF-*α*, and 14 (pg/mL) for G-CSF. The intra- and interassay coefficients of variation for the ELISA results were less than 10%.

### 2.7. Real-Time RT-PCR

Viral titers in lung tissues were measured by real-time RT-PCR using specific RSV primers. Briefly, RNA was isolated from lung tissues stabilized in RNAlater reagent using an RNeasy kit (Qiagen) according to the manufacturer's instructions. The isolated RNA was transcribed into cDNA by ReverTra Ace *α* (Toyobo Co. Ltd., Osaka, Japan) using the primer (5′-ATGGCTCTTAGCAAAGTCAAGTTG-3′) for the RSV N gene region according to the manufacturer's instructions. The RSV cDNA was amplified and analyzed on a Roche LightCycler P2000 real-time PCR machine using a Roche LightCycler FastStart DNA Master SYBR Green I kit (Roche Diagnostics, Indianapolis, IN, USA) with a pair of RSV-specific primers (forward primer: 5′-AGATCAACTTCTGTCATCCAGCAAATACACCAT-3′, reverse primer: 5′-TGTTTCTGCACATCATAATTAGGAGTATCAATA-3′). The amounts of RSV cDNA were determined by comparing the crossing point value of the cDNA sample to those of pWRSN-1, a TA vector harboring a part of the RSV N gene (nt. 1096–1347).

### 2.8. DNA Microarray Test

DNA microarray analysis of the lungs of mock- or RSV-infected mice was performed by Hokkaido System Science Co., Ltd. (Sapporo, Japan). Briefly, RNA was isolated from lung tissues using an RNeasy kit according to the manufacturer's instructions. After a quality check of the isolated RNA, cDNA was synthesized and amplified from the RNA sample using a Low Input Quick Amp Labeling Kit (Agilent Technologies, Santa Clara, CA, USA). Exhaustive analysis of the gene expression of the sample was performed using a SurePrint G3 Mouse GE 8 × 60 K 1 color system (Agilent Technologies). Significant changes in the gene expression were discovered and analyzed using the software program GeneSpring (Agilent Technologies).

### 2.9. Assay of IL-6 Production from J774.1 Cells

Subcultured J774.1 cells were suspended in RPMI-1640 medium supplemented with 2% heat-inactivated FCS, 100 units/mL of penicillin G, and 100 *μ*g/mL of streptomycin (maintenance medium). One hundred microliters of J774.1 cell suspension (1 × 10^5^ cells/mL) was seeded in each well in a 96-well microtiter plate and incubated at 37°C for 24 h in humidified air with 5% CO_2_. After incubation, the culture medium was removed by aspiration and replaced by maintenance medium with or without methamidophos at 10, 30, or 100 *μ*M. Following 24 h incubation, the cells were inoculated with RSV at three multiplicities of infection and then incubated at 37°C for 1 h. Following virus adsorption, each well was washed and maintenance medium with or without methamidophos was added. The plates were further incubated at 37°C for 3 d in humidified air with 5% CO_2_. After incubation, the culture supernatant was harvested from each well, and the amount of IL-6 was measured by ELISA.

### 2.10. Statistical Analysis

Comparisons of body weight, food consumption, and the levels of cytokines of the controls with experimental groups were carried out using Student's *t*-test. Student's *t*-test was also used for the result for the levels of IL-6 in the culture supernatant of J774.1 cells. A *P* value of 0.05 or less was considered to be significant.

## 3. Results

### 3.1. Toxicological Effects of Perinatal Exposure to Methamidophos

**Figure 1:**
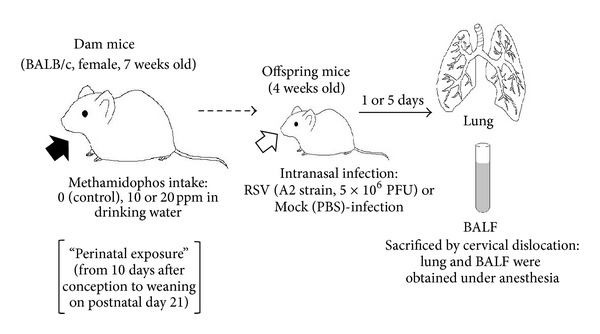
Schematic diagram of assay system.

Outline of the assay system was represented in Figure 1. In this study, general toxicological signs such as suppression of body weight gain and food consumption in dams and of body weight of offspring were monitored. Body weight of dams exposed to methamidophos was suppressed approximately 20% compared to the control (Table 1). Reduced food consumption was also observed in dams exposed to methamidophos at 20 ppm. However, no loss of body weight was detected in the offspring exposed perinatally to methamidophos (Table 1). From 5 to 8 offsprings were born to each dam in control and methamidophos-exposed groups. After weaning, no significant difference in the size of litters, survival rates, or food consumption of pups after weaning was detected between control and methamidophos-exposed groups (data not shown). No particular toxicological sign or abnormal behavior was observed in offspring mice. Then, offspring mice were infected intranasally with the A2 strain of RSV, and the following analyses were performed.

### 3.2. Effects of Perinatal Exposure to Methamidophos on Cytokine Production in RSV-Infected Offspring

To investigate the effects of perinatal exposure to methamidophos on the immune system of RSV-infected offspring, the levels of various cytokines in BALF were measured by ELISA on days 1 and 5 after infection (Table 2). In mock-infected offspring mice treated with or without methamidophos, the levels of cytokines in BALF were under the limit of detection (data not shown). On day 1 after infection, the levels of IL-6 in offspring mice exposed to methamidophos at 10 and 20 ppm were significantly decreased to approximately 66% (*P* < 0.05) and 50% (*P* < 0.01), respectively, of the control. But, the levels of TNF-*α* and G-CSF were suppressed in a dose-dependent manner, but not significant due to perinatal exposure to methamidophos. The levels of IL-1*β* and IL-12 were under the limit of detection. On day 5 after infection, the levels of IFN-*γ*, a representative marker of pneumonia due to RSV infection, in BALF from offsprings exposed to methamidophos were significantly (*P* < 0.05) decreased to approximately 54% of the control. Regarding Th2 cytokines, the levels of IL-10 were not significantly reduced due to perinatal exposure to methamidophos compared with the control, and those of IL-4 were under the limit of detection. Thus, perinatal exposure to methamidophos suppressed production of the proinflammatory cytokines IL-6 and IFN-*γ* in RSV-infected offspring mice.

### 3.3. Effect of Perinatal Exposure to Methamidophos on Severity of RSV Infection in RSV-Infected Offspring

**Figure 2:**
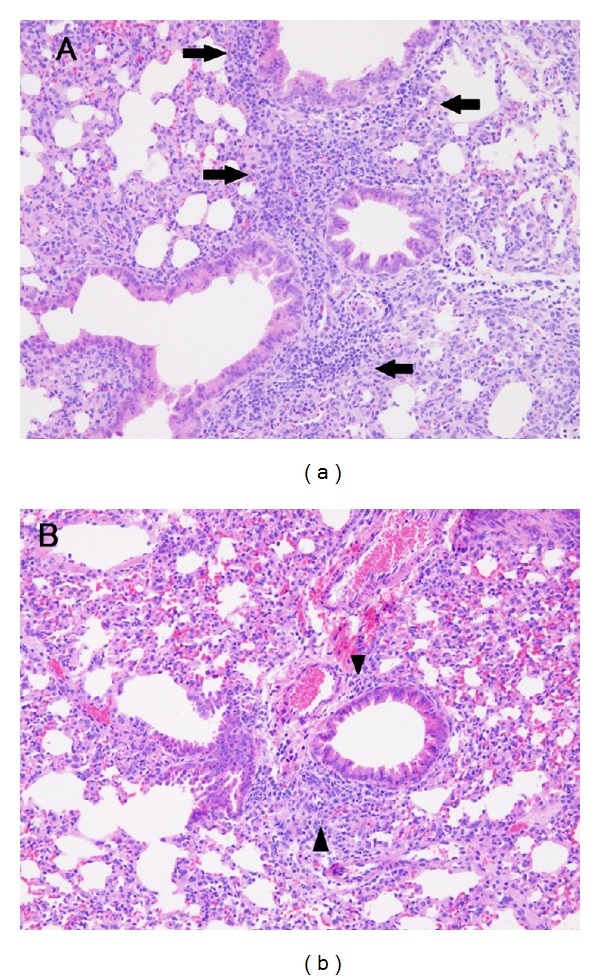
Lungs of mice 5 d after RSV infection. In this experiment, 6 mice per group were used, and representative data are shown. A, Control mouse with RSV infection. B, Methamidophos-treated (20 ppm) mouse with RSV infection. Marked changes in histological findings are indicated as follows: arrow, moderate infiltration of mononuclear cells; arrowhead, slight infiltration of mononuclear cells. Hematoxylin and eosin stain (×100).

The effect of methamidophos on the severity of RSV infection in mice was investigated. Viral titers of lung tissues in RSV-infected offspring on day 5 after infection were measured by real-time RT-PCR using the specific primer for RSV (Table 2). The viral titers in methamidophos-exposed offsprings were almost equivalent to those in the control. The effects of perinatal exposure to methamidophos on lung tissues of RSV-infected offspring mice were analyzed histopathologically (Figures 2(a) and 2(b)). While moderate peri bronchiolar inflammation was observed on day 5 after infection in the control mouse (Figure 2(a)), slight peri bronchiolar inflammation was found in the infected mouse treated with methamidophos (Figure 2(b)). Suppression of infiltration of mononuclear cells was typically observed (Figure 2(b)). No histopathological change was found in lung tissues of mock-infected offspring mice treated with or without methamidophos (data not shown). These results indicated that perinatal exposure to methamidophos did not affect the growth of RSV but suppressed moderately the expansion of the virus-induced pneumonia in offsprings.

### 3.4. Gene Expression in Lung Tissues of Offspring

Because effects of methamidophos on the immune system in RSV-infected offspring mice were observed, a change of the gene expression in lung tissues of offspring was sought by exhaustive analysis using a DNA microarray. We integrated the data of gene expressions that changed during RSV infection compared with mock infection and then changed due to methamidophos-exposure compared with the control. Data with a more than 2-fold change of gene expression were selected and, finally, 573 genes were determined. Ten genes related to the immune and/or inflammatory system were found among them (Table 3). Due to perinatal exposure to methamidophos, expressions of seven genes were suppressed. Particularly, the expression of IL-6 and IFN-*γ* genes was strongly suppressed to 24% and 34%, respectively. These results accorded almost with those of cytokine levels in BALF from RSV-infected offspring (Table 2). The gene expression of G-CSF was also suppressed to 29%. Then, the level of G-CSF in BALF was suppressed in a dose-dependent manner, but not statistically significant (Table 2). Changes of gene expression of both TNF-*α* and IL-10 were within 2-fold and not determined in this analysis, although the levels of those in BALF were quantitative by ELISA (Table 2). Changes of gene expression of IL-1, IL-4, and IL-12 in which their levels were under the limit of detection of ELISA in BALF (Table 2), were also within 2-fold and not determined in this analysis (Table 3). Thus, perinatal exposure to methamidophos affected the expression of specific cytokine genes in lung tissues of RSV-infected offspring mice.

### 3.5. Effects of Methamidophos on IL-6 Production by J774.1 Cells

**Figure 3:**
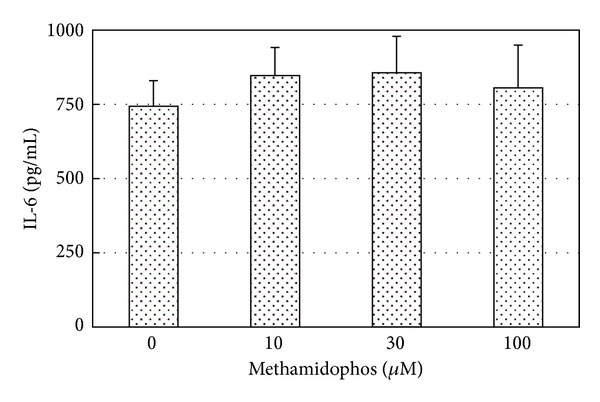
Effects of treatment of methamidophos on IL-6 production from RSV-infected J774.1 cells. J774.1 cells were incubated at 37°C for 24 h with or without methamidophos at 10, 30, or 100 *μ*M. After incubation, the cells were infected with RSV at three multiplicities of infection and further incubated with or without methamidophos. Following 3 d incubation, the culture supernatant was harvested and the amount of IL-6 was measured by ELISA. Data represent mean ± standard deviation (vertical bars).

We evaluated the suppressive effect of methamidophos on cytokine production using the macrophage-like cell line J774.1 (Figure 3). The cell line was established from BALB/c mouse and has been used to produce the proinflammatory cytokines due to some stimulation such as lipopolysaccharide stimulation and virus infection [11]. The levels of IL-6 in the culture supernatant of mock-infected J774.1 cells treated with or without methamidophos were under the limit of detection of ELISA (data not shown). Treatment with up to 100 *μ*M methamidophos did not suppress directly the production of IL-6 from RSV-infected J774.1 cells.

## 4. Discussion

Use of methamidophos, an organophosphate insecticide, is strictly regulated due to its severe neurotoxicity, but illegal use is suspected to cause contamination of agricultural foods in many countries. In addition, acephate, which belongs to the same category of insecticide, is used widely and converted to methamidophos by hydrolysis in animals [12]. The immunotoxicity of organophosphates is not known widely, but modulation of the immune system by organophosphates has been reported [13, 14]. We therefore evaluated the effects of exposure to methamidophos during development on the immune system response to a virus infection (Figure 1).

Before starting RSV infection, general toxicological effects of methamidophos were examined (Table 1). Suppression of body weights and food consumption was observed in dams, but no toxicological sign was seen in offspring mice. Rats were similarly exposed to methamidophos during gestation, and no particular toxicological signs including behavioral abnormality were observed in pups [15]. Prenatal exposure to methamidophos resulted in a transient decrease in cholinergic receptors, but they had returned to normal by 3-4 weeks of age in rats [16]. In our study, the contribution of methamidophos to toxicity in the nervous system might be slight in offspring mice.

In a previous study, we evaluated the effects of environmental contaminant brominated flame retardants (BFRs) on developmental immunotoxicity using an RSV infection mouse model and found that decabrominated diphenyl ether (DBDE) exacerbated pneumonia due to an increase in pulmonary viral titers and IFN-*γ* production in lung tissues [9]. Disorders of primary immunity, such as reduced productions of both TNF-*α* and IL-6, and increased those of IL-1*β*, were also obtained in RSV-infected offspring mice [10]. Although the level of IL-6 in BALF of RSV-infected offspring mice was significantly suppressed by methamidophos treatment, the level of IFN-*γ* and pulmonary viral titers were suppressed and not increased, respectively, (Table 2). In a histopathological analysis, pneumonia was moderately lessened due to perinatal exposure to methamidophos (Figure 2). Particularly, the inflammatory was marked by suppression of infiltration of mononuclear cells, such as lymphocyte and monocyte/macrophage (Figure 2(b)). This might be consisted in the low levels of the proinflammatory cytokines in BALF (Table 2), because IL-6 and IFN-*γ* activate T lymphocyte and macrophage, respectively.

To clarify the mechanism of action of methamidophos on the immune system in RSV-infected offspring mice, a DNA microarray test was performed on day 1 after infection (Table 3). It was verified that gene expressions of both IL-6 and IFN-*γ* were strongly suppressed in the lung tissues of RSV-infected offspring treated with methamidophos. Although gene expression of G-CSF was also suppressed, the results for the level of protein were not statistically significant (Table 2). This discrepancy may consist in the low levels of the cytokine in BALF. It is possible that methamidophos affected production of the cytokine in endothelial cells and monocytes/macrophages remaining in lung tissues of offsprings. Moreover, G-CSF may work in the initial phase of RSV infection, such as within 8 hours after infection. In a DNA microarray test, TNF-*α* and IL-10 were not selected (Table 3), although the levels of them in BALF were clearly detected in RSV-infected offspring (Table 2). Methamidophos might affect the process of RSV antigen and induced subsequently the suppression of production of TNF-*α* in macrophages [10]. As for IL-10, change of gene expression of the cytokine occurred probably in late phase responding to RSV infection. Suppression of gene expression of IL-18 receptor accessory protein might contribute to reduction of pneumonia in RSV-infected offspring mice treated with methamidophos (Table 3), because IL-18 is an inducer of IFN-*γ*. IFN-*γ* is a common marker of the progression of RSV-induced pneumonia in humans and mice [9, 10]. Although it is controversial whether the cytokine exacerbates the pneumonia [17], repression of progression of pneumonia due to methamidophos exposure might be due to suppression of IFN-*γ* production.

The *in vitro* experiment using J774.1 cell culture showed that methamidophos did not suppress RSV-induced production of IL-6, suggesting that the compound worked on the immune system in an indirect manner *in vivo* (Figure 3). Thus, perinatal exposure to methamidophos suppressed indirectly productions of proinflammatory cytokines due to reduction of their gene expressions, resulting in moderate repression of pneumonia without affecting the RSV growth in the lung tissues of RSV-infected offspring mice.

In contrast to environmental contaminant BFRs [9, 10], developmental exposure to methamidophos did not degenerate pneumonia and suppressed the production of the proinflammatory cytokines in response to RSV infection in the lung tissues in the offspring generation of mice. However, it is doubtful that methamidophos only affects the proinflammatory cytokines. Therefore, further studies on the other immune responses, including humoral immunity, are needed. Results for food contaminant methamidophos on immune system will be useful in the health sciences to manage risk in both mothers and children. Recently, an outbreak of food poisoning due to consumption of Chinese dumplings that had been intentionally contaminated by methamidophos was reported [18]. Thus, to protect the health of mothers and children from threatening environmental contaminants such as organophosphates, the mechanism of action of methamidophos on suppression of the proinflammatory cytokines presented in this study should be elucidated as soon as possible.

## 5. Conclusion

Perinatal exposure to methamidophos, a representative organophosphate insecticide, suppressed the production of proinflammatory cytokines in response to RSV infection in the lung tissues in the offspring generation of mice.

## Figures and Tables

**Table 1:** Body weights and food consumption of mice perinatally exposed to methamidophos.

Group/methamidophos (ppm)	Body weight (g)	Food consumption (g/week)
Dam		
0	28.7 ± 2.0	60.6 ± 11.5
10	22.1 ± 0.8**	59.4 ± 2.6
20	23.0 ± 2.0**	37.8 ± 12.4*
Offspring		
0	17.2 ± 1.1	0
10	16.3 ± 0.7	0
20	16.5 ± 1.7	0

The data represent mean ± standard deviation values of at least six mice. Body weight was assessed on postnatal day 21. Food consumption of each mouse was checked weekly and is expressed as a mean value ± standard deviation during methamidophos exposure.

*Significantly different from control at *P* < 0.05 (Student's *t*-test).

**Significantly different from control at *P* < 0.01 (Student's *t*-test).

**Table 2:** Effects of methamidophos on cytokine levels in BALF and pulmonary viral titers in RSV-infected offspring mice.

Methamidophos (ppm)	Day 1 after infection					Day 5 after infection			
	Cytokine level in BALF (ng/mL)					Cytokine level in BALF (ng/mL)			Pulmonary viral titer (copy/mL)
	TNF-*α*	IL-6	IL-1*β*	IL-12	G-CSF	IFN-*γ*	IL-4	IL-10	
0	0.83 (0.24)	0.50 (0.10)	<0.04	<0.08	0.08 (0.01)	1.50 (0.47)	<0.04	0.26 (0.13)	3.4 × 10^5^ (1.3 × 10^5^)
10	0.68 (0.28)	0.33* (0.02)	<0.04	<0.08	0.07 (0.01)	1.33 (0.78)	<0.04	0.25 (0.13)	—
20	0.53 (0.18)	0.25** (0.07)	<0.04	<0.08	0.06 (0.01)	0.81* (0.38)	<0.04	0.14 (0.02)	2.5 × 10^5^ (4.0 × 10^4^)

Data represent mean of values of 6 mice. Numbers in parentheses indicate standard deviation of values.

*Significantly different from control at *P* < 0.05 (Student's *t*-test).

**Significantly different from control at *P* < 0.01 (Student's *t*-test).

**Table 3:** Effects of perinatal exposure to methamidophos on gene expression in lung tissues of RSV-infected offspring mice by microarray analysis.

Fold change	Gene name	GenBank accession no.
4.50	Membrane-associated ring finger (C3HC4) 1	NM_175188
4.42	C-type lectin domain family 4, member a2	NM_001170333
3.26	Lipoic acid synthetase	AK013575
0.34	Interferon gamma	NM_008337
0.29	Colony stimulating factor 3 (granulocyte)	NM_009971
0.27	CD1d2 antigen	NM_007640
0.27	Interleukin 18 receptor accessory protein	NM_010553
0.24	Interleukin 6	NM_031168
0.22	Chemokine (C motif) ligand 1	NM_008510
0.14	Regenerating islet-derived 3 gamma	NM_011260

Data with more than 2-fold change in gene expression compared with RSV-infected control are included.

Ten genes related to the immune and/or inflammatory system were selected using the software GeneSpring.
